# Protein-Templated Metal Nanoclusters: Molecular-like Hybrids for Biosensing, Diagnostics and Pharmaceutics

**DOI:** 10.3390/molecules28145531

**Published:** 2023-07-20

**Authors:** Sherwin Chong Li Tan, Zhijian He, Guan Wang, Yong Yu, Le Yang

**Affiliations:** 1 Institute of Materials Research and Engineering (IMRE), Agency for Science, Technology and Research (A*STAR), 2 Fusionopolis Way, Innovis #08-03, Singapore 138634, Singapore; 2 Department of Materials Science and Engineering, College of Design and Engineering, National University of Singapore, 9 Engineering Drive 1, Singapore 117575, Singapore; 3 Institute of Sustainability for Chemicals, Energy and Environment (ISCE2), Agency for Science, Technology and Research (A*STAR), 2 Fusionopolis Way, Innovis #08-03, Singapore 138634, Singapore

**Keywords:** protein-templating synthesis, metal nanoclusters, nanohybrids, luminescence, core-shell structure, biosensors, bioimaging, therapeutics

## Abstract

The use of proteins as biomolecular templates to synthesize atomically precise metal nanoclusters has been gaining traction due to their appealing properties such as photoluminescence, good colloidal- and photostability and biocompatibility. The synergistic effect of using a protein scaffold and metal nanoclusters makes it especially attractive for biomedical applications. Unlike other reviews, we focus on proteins in general as the protective ligand for various metal nanoclusters and highlight their applications in the biomedical field. We first introduce the approaches and underlined principles in synthesizing protein-templated metal nanoclusters and summarize some of the typical proteins that have been used thus far. Afterwards, we highlight the key physicochemical properties and the characterization techniques commonly used for the size, structure and optical properties of protein-templated metal nanoclusters. We feature two case studies to illustrate the importance of combining these characterization techniques to elucidate the formation process of protein-templated metal nanoclusters. Lastly, we highlight the promising applications of protein-templated metal nanoclusters in three areas—biosensing, diagnostics and therapeutics.

## 1. Introduction

Ligand-protected, quantum-sized metal nanoclusters (NCs) consisting of tens to hundreds of metal (Au, Ag, Pt, Cu) atoms are ultrasmall particles (<3 nm in core) with attractive physicochemical properties [1,2,3,4,5]. The strong quantum confinement exerted on particles at this size regime allows them to possess molecular-like photoabsorptions and photoemissions [6,7,8,9], intriguing magnetism [10] and chirality [11,12,13,14,15,16,17] and excellent catalytic properties [18,19,20,21,22]. Among various ligand-protected metal NCs, protein-stabilized or protein-templated metal NCs are particularly interesting thanks to their unique protein-coated surface [23,24,25,26]. Proteins are large biomolecules or macromolecules with at least one long chain of amino acid residues in their primary structure which are further folded to form complex secondary and tertiary structures [27]. Due to their abundant surface functional groups, grooves and cavities hidden in their three-dimensional structures and strong steric hindrances, proteins are versatile to bind metal ions and direct the nucleating and growth of metal NCs similar to the well-known biomineralization process [28]. The resultant protein-templated metal NCs feature good colloidal stability, strong photoluminescence, good photostability, inherent biocompatibility and flexibility of post-synthesis modification, which are particularly attractive for diverse applications in biological systems.

It has been over 10 years since the first report of protein-templated synthesis of AuNCs by Xie et al. in 2009 [29]. In this seminal work, a globular protein named bovine serum albumin (referred to as its abbreviation BSA hereafter) with a molecular mass of 66.4 kilodaltons was used as a template. By activating the intrinsic reducing power of BSA at an elevated solution pH (11) and temperature (37 °C), an intensely red-emitting (λ_ex_ = 640 nm) AuNC was formed in a course of 24 h. Since then, numerous researchers have joined and expanded the scope of protein-templated metal NCs by studying the feasibility of synthesizing NC either with a different metal core or using various proteins, natural or synthetic, to tune the physicochemical properties of resulting metal NCs, or exploiting their applications in different biological settings. The high citation (>2000 times) of this article and emergence of hundreds of related research articles have proved the robustness of protein-templated synthesis of metal NCs and their great potential for many diverse applications. We noticed that despite the fact that a few nice review articles are out there which have summarized the progress of protein-templated metal NCs, they are either not focusing on protein as the protecting ligands [1,2,5,24,30,31] or restrict their discussion on certain specific aspects only [32,33,34,35,36]. One recent review article aimed to summarize the advances of protein-templated metal NCs, including the formation mechanisms during synthesis and relevant biological applications [37]. However, this work focuses on BSA-protected AuNCs only, which we believe may not be extensive enough to include all the interesting work in this field. More importantly, most of these review articles were published 5 years ago, which may not capture the most recent advancements in protein-templated metal NCs.

**Figure 1:**
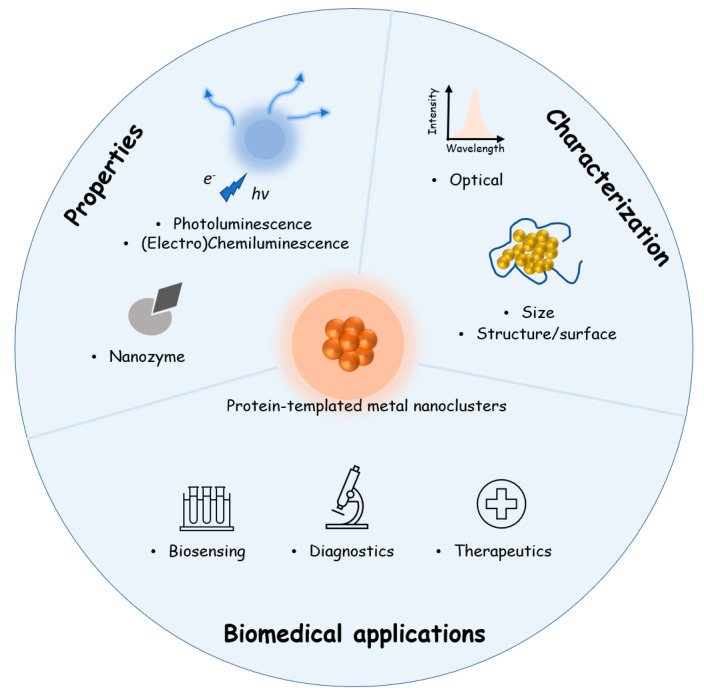
Schematic illustration of the three important aspects, including physicochemical properties, characterizations and biomedical applications of protein-templated metal NCs, as the pillars of protein-templated metal NCs built on the basis of the synthesis of protein-templated metal NCs.

In this contribution, we first review the synthesis approaches of protein-templated metal NCs with the emphasis on the source of reducing power. We then discuss the unique physicochemical properties of protein-templated metal NCs, particularly photoluminescence, electrogenerated chemiluminescence and nanozyme properties of different protein-templated metal NCs. Following that, the most important characterization techniques of protein-templated metal NCs are highlighted. Two case studies are given to demonstrate how these important characterizations are combined together to give valuable insights into the formation of protein-templated metal NCs and to guide the design of useful applications. Next, the biological applications of protein-templated metal NCs are summarized, including biosensing (categorized according to the mode of signal generation), diagnostics (including in vitro and in vivo imaging studies), drug/gene delivery and phototherapy (including photodynamic and photothermal therapies). As shown in Figure 1, the synthesis of protein-templated metal NCs forms the basis while the physicochemical properties, characterizations and biological applications are three important branches derived from the synthesized protein-templated metal NCs. This article cites previous studies strategically by not only picking the most typical examples but also the latest work, especially those published in the last three years. We think that this paper will provide an updated and complete review of the recent advances of protein-templated metal NCs for researchers working in the relevant area, especially those who are new to but eager to explore this exciting topic.

## 2. Synthesis Approaches of Protein-Templated Metal Nanoclusters

**Figure 2:**
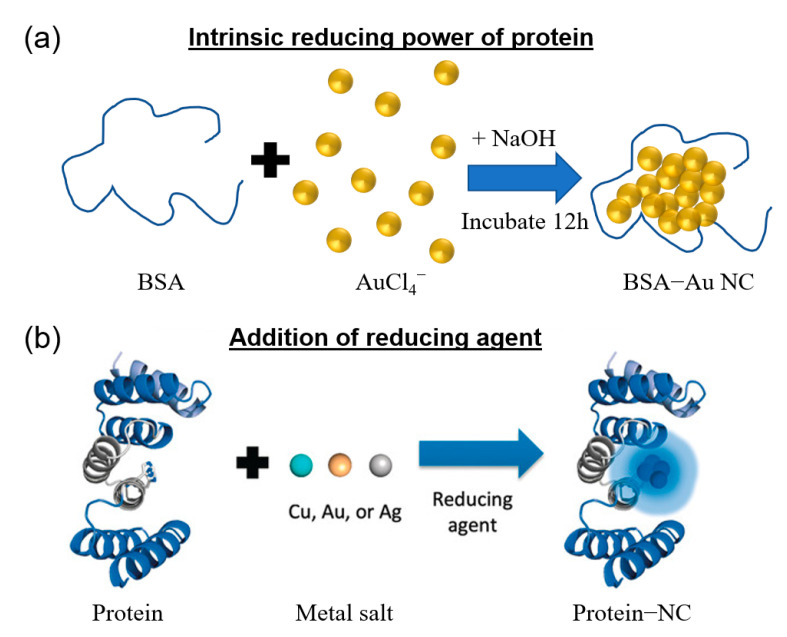
General approaches for the synthesis of protein-templated metal nanoclusters (NCs). Formation of NCs by (**a**) activating the reducing ability of the protein or (**b**) adding a reducing agent. Reproduced with permission from [46].

Protein templates offer the advantage of nucleating and stabilizing precise metal NCs. This is unlike non-templated synthesis which often results in products of various uncontrolled sizes. Protein-templated metal NCs are generally synthesized by mixing aqueous solutions of the metal salts and protein at room temperature and ambient pressure. In this template-assisted method, a suitable template with a predetermined structure is first used to bind with the metal ions, followed by reduction in the template to obtain metal NCs. As shown in Figure 2, the metal–protein complex can be reduced by either (a) activating the self-reducing ability of the protein or (b) introducing an additional reducing agent. The utilization of the intrinsic reducing ability of protein was originally described and best depicted in this 2009 paper, where a high solution pH activated the intrinsic reducing power of BSA protein [29]. Apart from pH, there are other methods such as heating, microwave or ultrasonic radiation that can be used to activate the reducing power of the protein. For example, several research groups have demonstrated that protein-templated metal NCs could be successfully synthesized using a microwave-assisted method [38,39,40,41]. Microwave-assisted synthesis allows more uniform heating and has been shown to significantly reduce reaction time from hours to tens of minutes [42]. In cases where the intrinsic reducing power of the protein itself is insufficient or absent, additional reducing agents have been used to successfully form different metal (Au [43], Cu [44], Ag [45]) NCs.

The difference in the approaches depicted in Figure 2 originates from the balance of nucleation and growth of metal NCs and the stabilization/protection provided by protein templates. The selection of an appropriate protein template is critical in the synthesis of NCs as protein molecules provide a unique coordination environment for the formation of metal NCs through their specific amino acid residues, unique secondary and tertiary structures and resulting cavity sizes. Proteins contain large numbers of electron-bearing groups such as hydroxyl, amine and carboxyl groups on their surface, which are capable of specific or non-specific adsorption to metal ions through electrostatic, complexation or chemical bonding interactions. Proteins which contain sulfhydryl groups are widely used in the synthesis of metal NCs due to strong interactions between metal ions and sulfhydryl groups. For instance, a thiol-bearing protein is promising for synthesizing AuNCs as it could form stable Au-S covalent bonds as demonstrated by the ample reports of thiolate-protected AuNCs [17,22,47,48].

The protein size and existence of cavities to accommodate the formation of metal NCs is one important consideration in its synthesis. The use of an unsuitable protein template can lead to ineffective protection of the metal NCs, resulting in metal NCs liberating from the protein scaffold and coalescing to form nonfluorescent clusters of even larger metal NPs [26]. For example, pepsin-protected Au NCs had significantly lower fluorescent intensity compared to BSA-protected Au NCs as pepsin only had a few amine groups which were insufficient for effective complexation with gold ions and stabilization of formed AuNCs [49]. In addition, the concentration of protein template also plays a part in affecting the size of metal NCs. A decrease in protein concentration would produce large non-fluorescent nanoparticles owing to the deficiency in protection of NCs [50]. The feasibility in controlling the size of the protein cavity is ideal to tuning the size of the formed metal NCs, which will be further discussed in Section 4.

Another important consideration for the selection of the protein template is its intrinsic reducing ability and the mechanism to trigger its reducing power. Given the fact that the ability of different metals to form complexes with protein are varied, designing a suitable synthetic strategy is needed for each specific synthesis. For instance, in most synthesis reactions, an alkaline environment is used to activate the reducing power of the protein as common proteins have a reducing ability due to the thiol groups on their surface (the intrinsic pK_a_ is at ~pH10–11) which can be used as both stabilizers or reducing agents during the formation of particular AuNCs [44]. However, there have been relatively fewer reports of protein-templated copper NCs (CuNCs) as compared to their Au and Ag analogues. Despite its attractiveness in applications in the fields of catalysis, medical diagnosis, therapy, chemical sensing and light-emitting devices [51,52], CuNCs are hard to form inside protein templates and are prone to oxidations. One possible reason is the variety of functional groups on the protein surface such as amine and carboxyl groups that can form strong coordination with Cu (II) to prevent their reduction and nucleation. Meanwhile the easy oxidation of metallic Cu requires better protection from the protein template. It has been shown that amine-bearing reducing agents such as hydrazine serve as a good reducing agent to form stable protein-templated CuNCs [44].

Based on the synthetic approach adopted, we summarize in Table 1 some of those typical proteins that have been reported to prepare metal NCs and their corresponding synthesis approaches. The utilized proteins with their molecular weights and physical properties (e.g., core size, photoluminescence color and quantum yield if any) as well as their respective applications are also listed for better reference for the discussions in the subsequent sections.

## 3. Physicochemical Properties of Protein-Templated Metal Nanoclusters

It is well established that the properties of metal NCs are very different compared to the traditional bulk metal. Due to their ultrasmall size, metal NCs have discrete electron energy levels which results in their unique optical, chemical, electronic and magnetic properties. The properties of the synthesized protein-templated metal NCs are affected by both the metal core and the surface protein species, which provides more possibilities and opportunities for regulating their properties [66,67]. In this section, we highlight and discuss the properties that are relevant for biomedical applications, including photoluminescence, chemiluminescence/electrochemiluminescence and enzymatic properties.

### 3.1. Photoluminescence Properties of Protein-Templated Metal Nanoclusters

**Figure 3:**
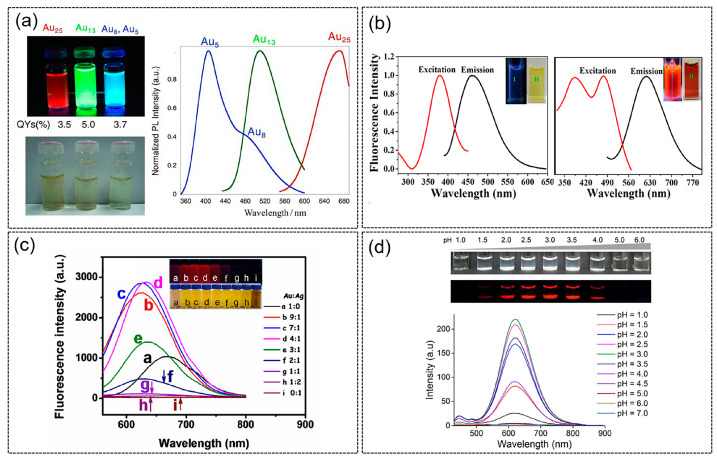
(**a**) Photographs and corresponding photoluminescence spectra of pepsin-mediated AuNCs with different emissions under UV light (above) or visible light (below). Reproduced with permission from [56]. (**b**) Fluorescence spectra of Ag_9_ NCs (left) and Ag_14_ NCs (right). Insert: photographs of respective AgNCs taken under UV (I) and visible light (II). Reproduced with permission from [75]. (**c**) Fluorescence emission spectra of Au-AgNCs with varying molar ratios. Insert: photographs under visible light (bottom) and UV light (top). Reproduced with permission from [76]. (**d**) Luminescence spectra of BSA-CuNCs in PBS at different pH values and their corresponding photographs under visible light (top panel) or UV light (lower panel). Reproduced with permission from [77].

Though metal NCs exhibit several molecular-like properties, their photoluminescent (PL) properties have always been of great interest due to their potential widespread applications as luminescence probes in the environmental and biological systems [30,68,69,70,71,72,73]. Generally, metal NCs exhibit strong absorption in the UV region while having a long absorption tail in the visible region. This is starkly different from the surface plasmon resonance (SPR) peak observed for larger (i.e., >3 nm) nanoparticles. The absorbance bands of metal NCs are the result of the discrete electronic states and strong quantum confinement effects. The photoluminescent properties of metal NCs are attractive for biomedical applications [74]. Compared with traditional fluorescent materials, such as organic dyes, semiconductor quantum dots and perovskite quantum dots, metal NCs exhibit better fluorescence properties, such as emission wavelength tunability and excellent photostability. For instance, by changing parameters such as pH or temperature during synthesis, the size and structure of protein-templated metal NCs can be changed, thereby allowing the tuning of photoluminescence properties. Through changing the core size of gold NCs by varying the pH during synthesis, Arakawa et al. [56] were able to achieve different wavelengths of fluorescence emissions, namely Au_5_/Au_8_ NCs with blue fluorescence emission, Au_13_NCs with green fluorescence emission and Au_25_ NCs with red fluorescence emission (Figure 3a). In addition, Mukherjee’s research group [75] synthesized Ag_9_ NCs with fluorescence emission wavelength at 480 nm and Ag_14_ NCs at 620 nm using human serum albumin (Figure 3b), with quantum yields of 16% and 11%, respectively. Furthermore, the blue- and red-emitting AgNCs could be converted to each other. Further reduction of the Ag_9_ NCs led to Ag_14_ NCs while the latter could be oxidized to the former.

Despite the advantages of utilizing proteins as templates for metal NCs, they have weak luminescence compared to the traditional quantum dots or fluorescent dyes. However, several strategies have been developed to improve the luminescence of metal NCs [37]. Recent studies have shown that the fluorescence properties of metal NCs can be effectively enhanced by incorporating other metals into single-metal NCs. Tang’s group synthesized Au-AgNCs by co-reducing gold-silver precursors with bovine serum albumin as reducing-cum-protecting agent [76]. As shown in Figure 3c, by varying the molar ratio of Au and Ag, fluorescence intensity that is significantly higher than that of single-metal AuNCs can be achieved due to the synergistic effect of Au and Ag. When the molar ratio of Au and Ag is 4:1, the fluorescence of Au-AgNCs is the strongest, and the emission wavelength is located at 630 nm.

Another strategy to enhance the photoluminescence property of NCs is to make use of the aggregation-induced emission (AIE) phenomenon. Recent studies have found that NCs similar to some small organic molecules also have AIE properties. Therefore, by adjusting the degree of aggregation of NCs, their fluorescence properties can be improved. In one example, Liu’s group synthesized BSA-protected copper NCs (Cu NCs@BSA) and achieved large fluorescence enhancement by leveraging on the AIE effect [78]. The aggregation behavior of the copper NCs was induced by adjusting the pH of the NC buffer solution (Figure 3d). In another example, Xie’s group used chitosan and glutathione-protected gold NCs to assemble and form a chitosan-gold nanocluster gel [77]. The favorable electrostatic attraction between chitosan and the thiolate ligands led to matrix-coordinate-induced aggregation resulting in improved photoluminescence efficiency. Polyvinyl alcohol (PVA) and Polyvinylpyrrolidone (PVP) have also been assembled with glutathione-protected copper NCs to improve the fluorescence quantum yield to over 30% by taking advantage of the AIE properties [79].

### 3.2. Chemiluminescence/Electrochemiluminescence of Protein-Templated Metal Nanoclusters

Chemiluminescence (CL) and electrochemiluminescence (ECL) are light emissions from excited states of a luminophore induced by chemical reactions. Unlike photoluminescence which requires an external excitation light source, CL/ECL is free from external light irradiation and, as a result, has advantages such as higher signal-to-noise ratio and improved sensitivity. The main difference between CL and ECL is that the latter requires an external potential applied at the electrode surface to trigger the electrochemical reactions.

**Figure 4:**
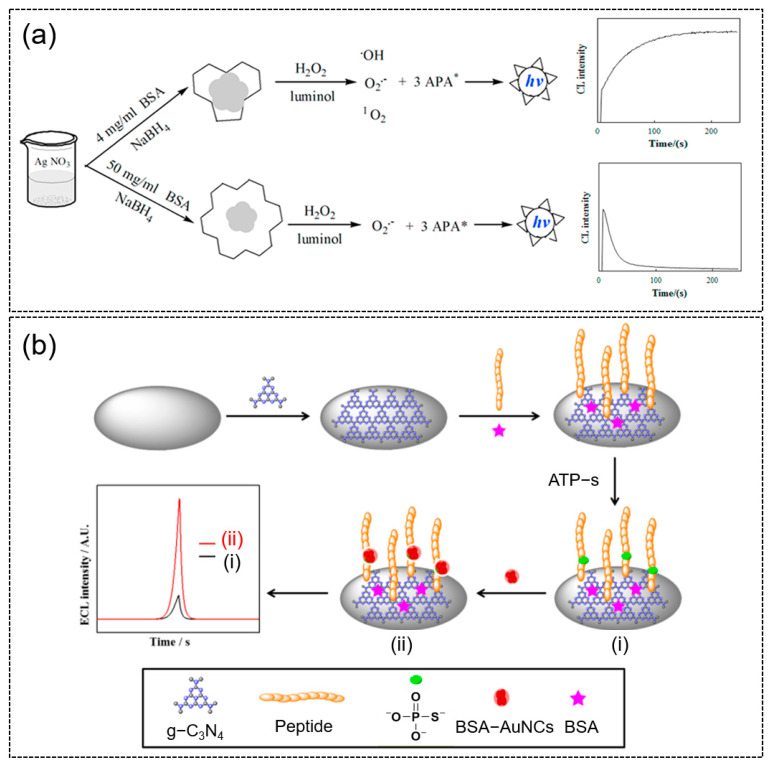
(**a**) Schematic of the chemiluminescence (CL) mechanisms of the luminol-H_2_O_2_ system catalyzed by AgNCs with different degrees of BSA templated and their respective CL kinetic curves. (Top) The generated •OH and O_2_^•−^ react with each other to form ^1^O_2_ which in turn reacts with luminol anion to produce aminophthalate anion (APA*). (Bottom) The generated superoxide anions react with luminol radicals to produce APA*. The subsequent relaxation of APA* to ground state emits light. Reproduced with permission from [84]. (**b**) Schematic of the ECL platform for the sensitive monitoring of PKA activity and the ECL intensity in the (i) absence and (ii) presence of BSA-AuNCs. Reproduced with permission from [93].

The CL phenomenon has been widely employed for various biomedical applications such as bioimaging [80,81], biosensing [82] and therapeutics [83]. The CL of protein-templated metal NCs is mainly from their role as catalysts, while their direct use as a luminophore or quencher is comparatively less. Protein-templated metal NCs have been reported to catalyze several CL systems such as the typical luminol-H_2_O_2_ [84,85,86] system and others including H_2_O_2_-fluorescein [87], luminol-NaIO_4_ [88] and K_3_Fe(CN)_6_-rhodamine 6G [89]. For example, Sheng et al. showed that BSA-templated AgNCs was able to efficiently catalyze the luminol-H_2_O_2_ CL reaction [84]. It was reported that the generated superoxide radical O_2_^•−^ played a critical role in the catalytic oxidation of the CL reaction. Interestingly, they also reported that by varying the capping degree of the BSA template on AgNCs, different CL kinetic curves can be obtained (Figure 4a). The group also successfully demonstrated the simple, rapid and sensitive detection of uric acid using the BSA-AgNCs catalyzed CL system.

Reports of using protein-templated metal NCs as luminophores or quenchers of CL systems are less than those utilizing the catalytic action of protein-templated metal NCs. In 2016, Li et al. designed a sensor platform based on CL resonance energy transfer for label-free detection of trypsin [90]. In their subsequent studies, the direct CL of BSA/AuNCs with various classic oxidants, such as KMnO_4_, N-bromosuccinimide, K_3_Fe(CN)_6_, H_2_O_2_ and Ce(IV), were also investigated [91]. They discovered that the acidic KMnO_4_-BSA/Au NCs CL reaction achieved the highest CL signal. As for reports on quenching the CL of protein-templated metal NCs, Vahid et al. reported that the CL intensity of the H_2_O_2_-HCO_3_^−^ system was increased with the introduction of CdSe quantum dots (QDs). They attributed the increase to the CL resonance energy transfer (CRET) between the CL emitters and CdSe QDs. Meanwhile, the catalytic activity of CdSe QDs also played a part [92]. BSA/AuNCs have been found to prohibit this CRET system and thus turn off the CL emission. The CL signal was subsequently recovered because of the leaching effect of cyanide on AuNCs. This strategy was exploited to develop a highly sensitive and reliable measurement of cyanide in environmental waters and biological samples.

On the other hand, ECL has also proven itself to be a powerful analytical tool. Both anodic and cathodic ECL applications of protein-templated metal NCs have been studied. Efforts in this domain have led to the discovery of versatile metal NCs that can be used as luminophores and catalysts, which provided more possibility for the discovery of novel and highly efficient ECL systems. Due to the good aqueous solubility, excellent colloidal stability, ease of synthesis and modification, protein-templated AuNCs have become the most investigated protein-templated metal NCs used for ECL applications. When used as a biosensor, studies have shown that the electrocatalytic effect of AuNCs on luminol oxidation can lead to a several-fold increase in ECL emission [94]. For instance, Luo et al. designed an ECL biosensor system in which BSA/AuNCs were used to enhance the cathodic ECL of graphite-like carbon nitride material (g-C_3_N_4_)-S_2_O_8_^2−^ and achieved the sensitive detection of protein kinase activity (PKA) (Figure 4b) [93]. The assembled BSA/AuNCs act as the catalysts of the ECL reaction, and the resultant ECL signal is magnified 4.5 times as compared to the scenario in the absence of BSA/AuNCs. The proposed ECL platform can analyze the PKA activity of biological samples quantitatively and screening kinase inhibition qualitatively. Apart from protein-templated AuNCs, the ECL properties of other protein-templated metal NCs such as Ag [84], Cu [95] and Ni [96] have also been explored.

An obstacle to the improvement of ECL performance is the unclear understanding of the ECL mechanism, and this has hindered the innovation of new protein-templated metal NCs. Efforts to increase ECL efficiency have also been met with limited success. Nevertheless, several general strategies have been explored to improve ECL efficiency—for example, the use of coreaction accelerators such as nanoparticles or active micro molecules to improve the ECL emission by effectively reducing the coreactant to a stronger oxidative or reductive intermediate radical [97]. In addition, to achieve more efficient intramolecular electron transfer, researchers covalently linked luminophores and coreactants or luminophores, coreactants and coreaction accelerators, as opposed to the original inefficient intermolecular electron transfer. It is also worth mentioning that both the ECL intensity [98] and the ECL wavelength [99] could be affected by the valence states of metal NCs. Lastly, the poor conductivity of protein coated on an NC surface could also limit mass transport and electron transfer, lowering their ECL efficiency and restricting potential applications [100].

### 3.3. Nanozyme Properties of Protein-Templated Metal Nanoclusters

Nanozymes have received extensive research attention due to their high catalytic efficiency, stability, economy and large-scale preparation. They are characterized by their unique physical and chemical properties in addition to their catalytic function. An early report found that BSA-templated red or blue emitting gold NCs showed peroxidase mimicking property under visible light irradiation [101]. The enzyme memetic BSA-AuNC was reasoned as a result of photogenerated carriers (e.g., electron or hole) reacting with oxygen or water molecules to form reactive species such as •OH and O_2_^•−^. The intrinsic enzyme mimicking activity of BSA-AuNC was further utilized to construct a colorimetric assay of trypsin and achieved limit of detection as low as 0.6 μg/mL. In 2020, Liu’s group reported that histidine-based self-assembling polypeptide nanozymes have oxidase-like activity [102], which can catalyze H_2_O_2_ efficiently through the formation of reactive ternary complex intermediates. At the same time, the supramolecular catalysts exhibiting the highest activity can be switched between inactive and active states without losing their activity during ten cycles of heating/cooling or acidification/neutralization treatment. More recently, protein-templated platinum [103] and osmium [104] NCs were reported to show intrinsic peroxidase-like activity. Nanozyme activities of Ir clusters formed in bilirubin oxidase, glucose oxidase and laccase were also reported [105]. Interestingly, the intrinsic enzymatic properties of these enzyme templates do not appear to be affected, and this is likely due to their mild synthesis conditions.

## 4. Characterization Methods for Protein-Templated Metal Nanoclusters

**Figure 5:**
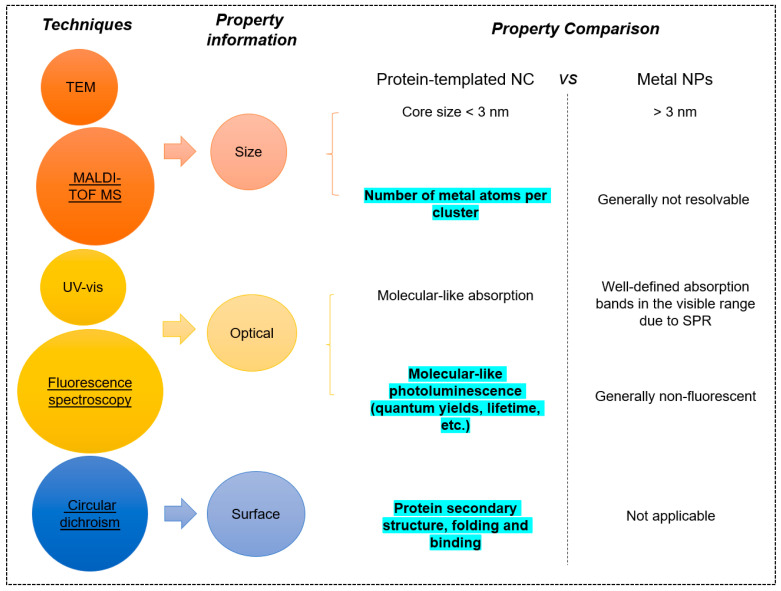
Essential characterization tools used to study protein-templated metal NCs and the useful information obtained using each technique. The most suitable techniques are underlined with the property information highlighted.

Protein-templated metal NCs feature ultrasmall sizes (<3 nm), discrete electronic structures, molecular-like photo absorptions and emissions and biocompatibility inherited from the protein shell [56,106,107,108,109,110,111]. Such characteristic features distinguish protein-templated metal NCs from not only their larger counterparts (i.e., metal NPs > 3 nm) but also their analogues protected by other different organic ligands (e.g., thiolates, DNA, etc.). Therefore, unique characterization techniques are required to better depict their properties, specifically the core size, photoluminescence and the structural information of selected proteins that are of most interest. Figure 5 lists the non-exhaustive but most useful techniques to characterize protein-templated metal NCs and the information that can be inferred from each method. After a brief introduction of these techniques, several case studies are presented to demonstrate how these methods are utilized to gain insights about the protein-templated metal NCs.

Transmission electron microscopy (TEM) has proved to be a well-established method to learn size information of inorganic and organic nanomaterials. However, TEM may not be sufficiently powerful to determine the sub-nanometer size of metal NCs. Apart from the compromised resolution, TEM is also known for resulting in structural changes in the NCs due to the heating effect of the electron beam [112]. Dynamic light scattering (DLS) is a commonly used technique to evaluate the hydrodynamic diameter of NCs [113,114]. The hydrodynamic size of the NCs has several biological significances—it affects the clearance, accumulation and uptake of the metal NCs in the body. For example, clusters that range from 3 to 6 nm are readily cleared from the kidneys while those less than 5 nm are able to rapidly transverse the walls of blood vessels [115]. However, DLS measurement only gives overall hydrodynamic size and cannot determine the core size of metal NCs, thus failing to differentiate between metal NCs and metal NPs.

Mass spectrometry (MS) is a more suitable analytic technique for ultrasmall NCs comprising tens to hundreds of metal atoms that resemble molecular entities [5]. Particularly, matrix-assisted laser desorption/ionization (MALDI) mass spectrometry has emerged as a reliable analytical technique for protein-templated metal NCs. MALDI is a soft and minimally destructive ionization technique as additional matrix molecules are introduced to absorb the laser radiation and transfer protons to NC. In combination with the time-of-flight (TOF) method, the cluster core size (number of metal atoms) and composition can be accurately determined [116,117].

UV-vis absorption and fluorescence spectroscopy have been widely used to characterize the absorption and emission profiles of metal NCs, respectively. Unlike larger metal NPs (typically > 3 nm) which exhibit well-defined absorption bands in the visible range (due to pseudo-continuous electronic structure and, thus, localized surface plasma resonance), protein-templated metal NCs only show a featureless absorption profile on the UV-vis spectra [29,45]. In addition, photoluminescence is an intriguing property of metal NCs due to electronic transitions between the occupied d bands and electronic states above the fermi level. Recent studies shed light on how the ligands used to stabilize the metal core can affect the luminescence of NCs through electron-rich atoms/functional groups which can transfer the charge to the metal core [66,110,111,118]. In the case of proteins with rich surface chemistries conferred by the abundant functional groups, the choice of protein template can also affect their luminescence properties. For example, gold NCs with the same number of core metal atoms can have varied PL properties depending on their capping protein. When protected by BSA, the Au_25_@BSA was found to have excitation peaks of 480 nm, emission maxima of 640 nm and QY of 6% [29], and when the protecting ligand was pepsin, the corresponding photophysical properties were changed to 360 nm, 670 nm and 3.5% [56]. In another example, the synthesis of gold NCs with different protein templates (lysozyme, HSA, BSA and gamma globulin) exhibited similar emission at 650 nm but had QY ranging from 3.8 to 5.4% [119]. Fluorescence spectroscopy is a useful analytical tool to identify the characteristic emission peaks of protein-templated metal NCs. Both steady state and transient state measurements can be done for absorption/emission spectroscopy, which not only give optical properties at equilibrium (e.g., how efficiently the NCs convert the absorbed energy into emitted light or quantum yield [120]) but also the dynamic processes of metal NCs in the excited states (e.g., how fast the photons at excited states return to the ground states [121]).

The protecting protein layer is unique for protein-templated metal NCs. They play several important roles for the formed metal NCs. First, protein templates provide sufficient protection to stabilize the nucleated NCs which possess only a few to tens of atoms and are highly active and instable. Second, proteins adopt specific three-dimensional structures which confer on their physiological functions. The grooves and cavities of protein can be utilized to control the size of as-formed metal NCs. In addition, protein can also influence the PL properties depending on the electron-donating/withdrawing ability in its functional groups [66,118]. Therefore, it is of interest to investigate the changes in structure after the integration of protein templates and metal NCs. Xu et al. did a comprehensive review on the influence of four selected model proteins (BSA, trypsin, pepsin and lysozyme) on the fluorescence of Au NCs [49]. It was discovered that the size of protein templates had major impacts on the stability of Au NCs. Furthermore, proteins with low cysteine contents were found to result in a blue emission (shorter wavelengths). Circular dichroism (CD) spectroscopy, an analytical tool measuring the differential absorption by the protein-templated metal NCs of right and left circularly polarized light, has demonstrated its versatility in studying the secondary structure of proteins and its folding and binding property [122].

In real cases, proper experimental designs and various different characterization techniques are needed to complement each other to obtain a better understanding of the protein-templated metal NCs. Below are two case studies of using the characterization tools described above to learn deeper or generate new knowledge of protein-templated metal NCs.

### 4.1. Case Study 1—The Origin of the Blue Emission in Protein-Templated AuNCs

**Figure 6:**
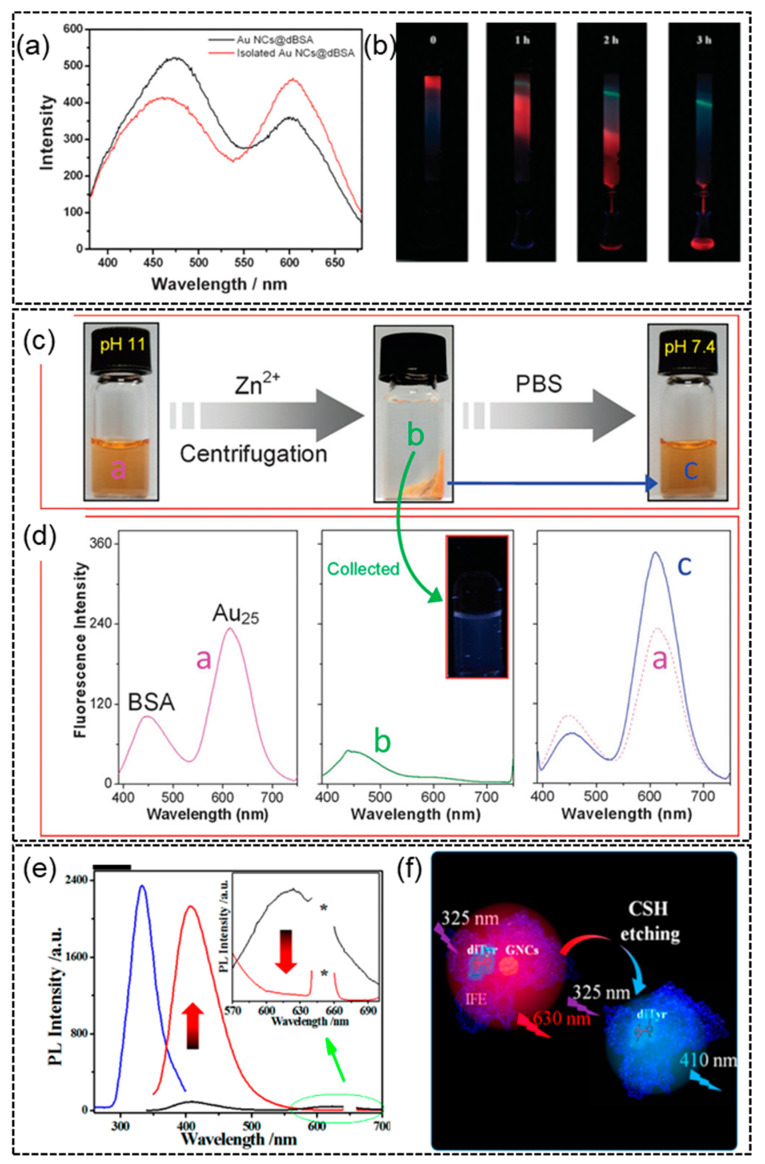
(**a**) Two emission bands of BSA-templated AuNCs and (**b**) visual gel separation of the blue- and red-emissive entities. Reproduced with permission from [125]. (**c**) Ion-induced separation-purification of BSA-templated AuNCs and (**d**) fluorescence spectra of the pre-centrifuged BSA-templated AuNCs, the supernatant after ion-nduced separation-purification and precipitated purified AuNCs after redispersion. a, b, and c represent as-synthesized BSA-templated AuNCs, the supernatant after centrifugation, and the precipitate redispersed in PBS buffer, respectively. Reproduced with permission from [126]. (**e**) Fluorescence spectra of the BSA-AuNCs before (black) and after (red) etching with cysteamine and (**f**) schematic illustration of the di-tyrosine residue in BSA-templated AuNCs. Blue trace represents excitation spectrum of the resultant solution (λ_em_ = 410 nm). The * marks the peak arises from second-order Rayleigh scattering. Reproduced with permission from [127].

The first case study is the long mysterious blue emission observed in BSA-templated AuNCs. Since the first report of BSA-templated AuNCs in 2009 [29], a blue emitter was observed together with the synthesized red-emissive BSA-templated AuNCs by many different research groups (Figure 6a, the emission peak near 450 nm). The blue emission was assumed to be from BSA protein due to tryptophan (Trp) and tyrosine (Tyr) residues in its sequence [123], and the emission of BSA was found to be concentration and/or aggregation-dependent [124]. A visual gel-separation method was designed to separate the unreacted BSA template from the formed BSA-templated AuNCs (Figure 6b) [125]. The relative abundance of the red emitter was increased after gel separation. However, the blue emission still remained. Similar results were reported in a separate study adopting an ion-induced precipitation separation method to purify the BSA-templated AuNCs (Figure 6c,d) [126]. Whether there were any interactions between the blue emitter and red BSA-AuNCs had been unknown. A more recent study used a strong etching agent (cysteamine) to etch away the red-emissive BSA-AuNCs and discovered that the blue emission was greatly boosted [127]. It was proposed that the blue emission was originated from the hidden di-tyrosine residues, which were formed by oxidation of gold chloride salts and initially masked by the red emission of formed BSA-AuNCs due to inner filter effect (IFE) (Figure 6e,f). Although the blue emission may not be from the di-tyrosine residues only, these reports do give us a deeper understanding of the formation process of BSA-templated AuNCs through proper experimental investigation.

### 4.2. Case Study 2—Protein Structure Change and Unfolding/Binding during Formation of AuNCs

**Figure 7:**
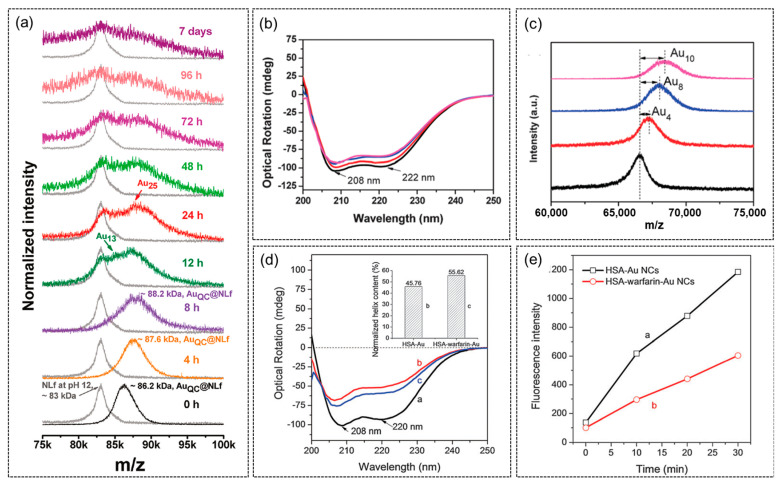
(**a**) Monitoring the time-dependent formation of AuNC inside native lactoferrin protein. Reproduced with permission from [128]. (**b**) Far-UV CD spectra and (**c**) MALDI-TOF mass spectra of AuNCs formed in BSA protein using a CO-reduction method. Black traces in (**b**,**c**) represent results from native BSA protein while red, blue, and magenta ones represent BSA-Au_4_, BSA-Au_8_, and BSA-Au_10_, respectively. Reproduced with permission from [43]. (**d**) Time-dependent PL and (**e**) far-UV CD spectra of HSA and drug (warfarin) bound HSA templated AuNCs. Black, red, and blue traces in (**d**) represent CD spectrum of HSA, HSA-AuNC, and HAS-warfarin-Au NCs, respectively. Reproduced with permission from [129].

As discussed earlier, MALDI-TOF MS and CD spectroscopy are analytical tools particularly useful for the characterization of protein-templated AuNCs. Here, several typical examples are collected to demonstrate the use of these characterization methods to study protein-templated AuNCs. Figure 7a shows the MALDI-TOF mass spectra of AuNCs formed inside native lactoferrin protein (NLf) [128]. The native protein gives a peak centered at ~83 kDa at pH12 on the mass spectrum. Upon the addition of Au ions, this peak shifted to ~86.2 kDa, indicating binding of ~16 Au ions. This peak further shifted to ~88.2 kDa after 8 h and was accompanied by the gradual increase of red emission, indicating the time-dependent formation of red-emitting Au_25_NCs. Furthermore, MALDI-TOF MS can also be combined with CD spectroscopy to establish a correlation between protein unfolding and the core size (number of Au atoms) in the resultant AuNCs [43]. The far-UV CD spectrum of BSA shows two characteristic peaks due to its α-helix structure (Figure 7b). It was discovered that the α-helix structure underwent a loss in feature when more Au atoms were confined in the resulting AuNCs, as identified through MALDI-TOF mass spectra (Figure 7c). The α-helix content is not only affected by the size of the formed AuNCs but also by the binding of small molecules. In a separate study [129], the binding of small drug molecule resulted in subtle changes of the helical structure of HSA (human serum albumin, with a similar structure to BSA) (Figure 7d). Using the drug-bound HSA as template while other synthesis conditions remained the same, the formation of AuNCs was slower than the unbound one, as monitored with the time-dependent evolution of fluorescence spectra (Figure 7e). This unique binding-formation kinetics correlation was further utilized for drug screening, as different drug molecules possess varied binding strengths.

## 5. Biomedical Applications of Protein-Templated Metal Nanoparticles

Protein-templated metal NCs consist of a metal core and protein as the ligand shell. As the outer shell of the metal NC is in direct contact with biological systems, using proteins as a biogenic ligand shell confers excellent biocompatibility to the metal NCs. Hence, utilizing proteins for preparing metal NCs, the unique optical and electronic properties of NCs could be combined with the inherent biocompatibility of the protein-surrounded complex to create remarkable synergistic effects which are ideal for biomedical applications. The unique advantages pertaining to protein-templated NCs are not only limited to improved biocompatibility but also include excellent water solubility and stability as reported in numerous works. Moreover, the rich surface chemistry and the large surface area-to-volume ratio of template-assisted metal NCs allows for the design of multifunctional nanomaterials for applications in biosensing, bioimaging and therapy [130]. The wide range of biomedical applications of protein-templated metal NCs are discussed in the following paragraphs.

### 5.1. Protein-Templated Metal Nanoparticles for Biosensing

**Figure 8:**
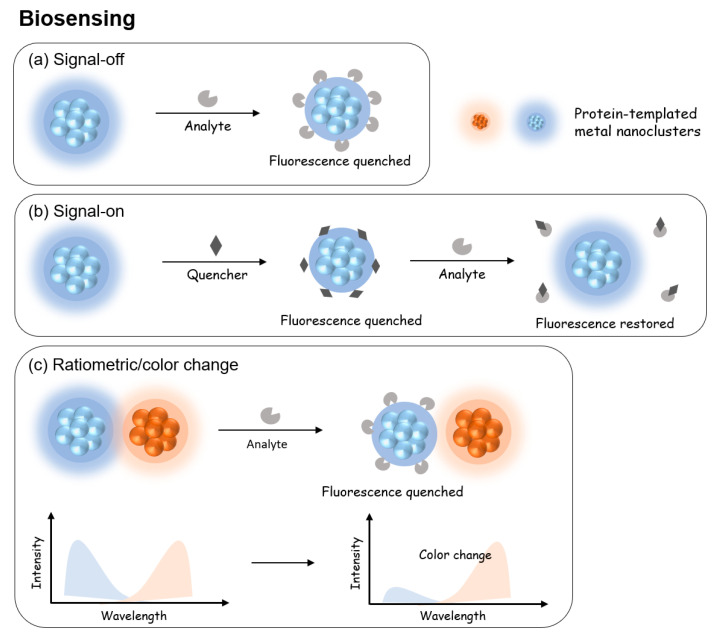
Schematic of the different signal generation approaches of protein-templated metal NCs for biosensing applications. (**a**) In the signal-off strategy, the fluorescence of metal NCs is quenched in the presence of analyte. (**b**) In the signal-on approach, the fluorescence of metal NCs is initially quenched, and upon presence of analyte, the analyte and quencher bind together preferentially, leading to the restoration of fluorescence. (**c**) In a typical ratiometric/color change strategy, two metal NCs probes with unique emission wavelengths are used. In the presence of the analyte, the fluorescence of one metal NC is quenched, leading to a color change or an emission of different intensity.

Protein-templated NCs have widely been used in the detection of pH [131,132,133], ions in biological systems [134,135,136] and biomolecules [137,138,139]. Such sensing is made possible by the specific interaction of the analyte with either the core or surface layer of protein-templated metal NCs, leading to fluorescence quenching, recovery or emission color change. Typically, three signal generation approaches, i.e., “signal-off”, “signal-on”, and “ratiometric”, have been developed for biosensing applications (Figure 8). In this section, the common strategies for biosensing are described, and the applications of NCs as a biosensor are highlighted. Table 2 summarizes some of the most recent reports of utilizing protein-templated metal NCs for applications within the field of biosensing, which are categorized according to their modes of signal generation. Important information such as the proteins used, the type of metal core, their optical properties such as excitation and emission wavelengths, photoluminescence quantum yield, target analyte, medium and sensor performance are also collated in detail.

#### 5.1.1. Signal-Off

In a signal-off strategy (Figure 8a), the analyte either interacts with the protein layer to deprive its protective role of the metal core or directly interacts with the metal core to break it down or change its size and/or structure. In either case, the initial fluorescence of the NC will disappear with the extent proportional to the amount of analyte. In essence, signal-off biosensing works on the principle of fluorescence quenching.

Protein-templated metal NCs for biosensors working on a signal-off mechanism are made possible due to their abundant functional groups (e.g., carboxyl, amine, sulfhydryl) providing readily available binding sites for quenching agents, most commonly metal ions. For example, Chen et al. successfully synthesized ovalbumin-stabilized gold NCs (OVA@AuNCs) and demonstrated its high sensitivity and selectivity for copper ions based on fluorescence quenching mechanism [140]. In the presence of Cu^2+^, the red emission of OVA@AuNCs was significantly quenched. The fluorescent probe demonstrated good linear correlation between the quenched fluorescence intensity and concentration of Cu^2+^ from 5.0 to 100 μmol/L and had a limit of detection of 640 nmol/L. As with any biosensor/fluorescent probe, regardless of its mode of detection, its usefulness depends on its selectivity towards a particular target. In this example, the OVA@AuNCs had high selectivity for Cu^2+^ while being relatively unaffected by other common metal ions. This was explained through the specific binding between copper ions and the histidine residue of the ovalbumin template which was also reported in another work [141]. The addition of Cu^2+^ led to the formation of stable metal complexes with the electron-rich functional groups of ovalbumin such as the carboxyl, amine and thiol groups. The formation of such complexes reduced the electron-donating effect of the ovalbumin template, leading to the fluorescence quenching of the OVA@AuNCs.

#### 5.1.2. Signal-On

Generally, the detection mechanism in signal-on biosensing is based on restoring the quenched fluorescence of metal NCs [138,142,143,144]. In this strategy (Figure 8b), the initial PL of NC will first be quenched by introducing a mediator quencher. In the presence of the target analyte, the quencher interacts with the analyte more easily, leading to the recovery of PL of the NC. This strategy utilizes the competitive interaction of the analyte with the quenching mediator over the NC probe. As a typical example, Nebu et al. successfully synthesized BSA-stabilized AuNCs that can detect cysteine/homocysteine (Cys/HCy) via signal-on of the fluorescence probe [138]. The synthesized BSA-stabilized AuNCs was initially quenched by KI_3_ due to the aggregation of the AuNCs from the S-S bond formation induced by I_3_^−^. In the presence of Cys/HCy, the thiol groups disrupt the S-S bond between the AuNCs and switch on the fluorescence.

#### 5.1.3. Ratiometric

Apart from fluorescence quenching and enhancement, another strategy for biosensing is based on ratiometric fluorescence (RF)/color change [145,146,147]. In this strategy (Figure 8c), the protein-templated metal NC is typically used in conjunction with a secondary probe emitting at a different wavelength and, thus, a different emission color [133,148,149]. The analyte interacts with the protein-templated metal NC to quench its emission, leaving only the emission of the second emitter. The extent of color change is typically represented as the ratio of two emission wavelengths and proportionally correlated to the amount of analyte. For example, Wang et al. developed an RF probe using BSA modified bimetallic gold/silver NC (BSA@Au/Ag-NC) for the detection of uric acid in blood samples [148]. The RF probe comprises the BSA@Au/Ag-NC, horseradish peroxidase (HRP) and o-phenylenediamine (OPD). Hydrogen peroxide (H_2_O_2_) is formed when uric acid is metabolized by the enzyme uricase. In the presence of H_2_O_2,_ HRP catalyzes OPD into 2,3-diaminophenazine (DAP) which quenches the fluorescence of BSA@Au/Ag-NC at 690 nm and concurrently increases the fluorescence of DAP at 580 nm. The RF-probe system demonstrated excellent linear correlation (R^2^ = 0.993) and remarkable accuracy. The development of simple and sensitive RF-probe for uric acid detection is attractive as serum uric acid is an important indicator of human health [150]. Furthermore, the RF-probe system can be easily modified to construct biosensors detecting other biomarkers such as glucose and lactate that are able to undergo reactions to produce H_2_O_2_.

While MNCs have attracted much attention as fluorescent probes for biosensing due to their good photostability and biocompatibility, it is worth noting that the PL quantum yields of metal NCs fall short of that of QDs. However, some groups were able to successfully create ultrabright MNCs by synthesizing bimetallic NCs as opposed to their mono metallic NCs counterparts [134,151]. In addition, several groups have utilized the well-known aggregation-induced emission (AIE) strategy to enhance the fluorescence of NCs [152,153,154].

**Table 2:** Summary of photophysical properties and biosensing applications of metal nanoclusters with protein ligand shell.

Sensing Principle	Protein	Metal	Optical Property ex/em, QY	Size (nm)	Analyte	Sample	Linear Range	LOD	Ref.
Signal off	Aprotinin	Gold	550/640 nm, 5.3%	2.84	Trypsin/mercury/ copper	Nil	0–150 μg/mL	10.18 μg/mL	[155]
Signal off	Amylase	Gold	383/660 nm, 7.9%	1.75	Deltamethrin/ Glutathione	Water/urine and plasma	0.01–5 μM/0.05–5 μM	6/10 nM	[156]
Signal off	BSA	Gold	450/650 nm, 8%	4–6	L-dopamine	Cerebrospinal fluid (CSF)	0–10 nM	0.622 nM	[137]
Signal off	BSA	Gold	365/600 nm	~2	Uric acid	Blood	0.7–80 μM	120 nM	[157]
Signal off	BSA	bimetallic gold-silver	270/630 nm	1.9	PPase activity	Bioassay for enzyme activity	0.1–30 mU/mL	0.03 mU/mL	[76]
Signal off	BSA	bimetallic gold-silver	370/620 nm	4.5	Hg^2+^ Cu^2+^	Blood samples	1.0–2000 nM 2.0–2500 nM	0.30 nM 0.30 nM	[134]
Signal off	BSA	Copper	320/405 nm	3	Fe^3+^	Wastewater and human blood serum	0.2–2.4 μM	10 nM	[158]
Signal off	HSA	Copper	325/405 nm	3 ± 0.3	bilirubin	Human urine and blood serum	Two linear range: 1.25–7.50 μM; 5.00–28.75 μM	35.0 nM 145 nM	[159]
Signal off	Lysozyme	Gold	370/650 nm, 5.2%	4	CN^−^	Nil	5–120 μM	0.19 μM	[160]
Signal off	Ovalbumin	Gold	470/630 nm	3.8	Cu^2+^	Serum	5.0–100.0 μM	640.0 nM	[140]
Signal off	Pepsin	Gold	416/655 nm, 7.4%	2	Spermine	Plasma & urine	0.0075–10 μM	1.75 nM	[161]
Signal on	BSA	Gold	370/610 nm, 6%	1.95	Alkaline phosphatase	Human serum plasma	1.0–200.0 U/L	0.05 U/L	[144]
Signal on	BSA	Gold	480/640 nm	4	Cysteine/ Homocysteine	Serum	0.0057–5 μM/8–25 μM	9 nM/12 nM	[138]
Signal on	BSA	Copper	325/406 nm	2.5	Dopamine	Urine samples	0.5 to 50 μM	0.28μM	[139]
Signal on	Chicken egg ovalbumin	Gold	370/640 nm, 6.6%	2.6	ATP/PPI	Serum	42–324 μΜ/9–70 μM	19 μM/5 μM	[42]
Signal on	Human serum albumin	Copper	325/405 nm	-	Human serum albumin	Serum and urine	~0.03–0.50 g L^−1^	1.8 ± 0.1 mg L^−1^	[53]
Signal on	Papain	Gold	490/639 nm	5.7	D-penicillamine	Rat serum	30.0 μM–2.0 mM	5.0 μM	[143]
Signal on	Papaya juice (papain, chymopapain)	Gold	360/440 nm	6.9	L-lysine	Urine	10.0–1000.0 μM	6.0 μM	[162]
Signal on	Transferrin	Gold	382/663 nm	2.2	5-HT	Human serum	0.2–50 μM	0.049 μM	[142]
Ratiometric	BSA	Gold	365/615 nm	~2	H_2_O_2_	Blood	0.05–10 μM	7.7 nM	[148,149]
Ratiometric	BSA	Bimetallic gold/silver	275/690 nm	5	Uric acid	Blood	5.0–50 μM	5.1 μM	[148]

### 5.2. Diagnostics

Biological imaging using fluorescent probes has always been of great interest for diagnostic and molecular biology purposes. Fluorescent metal NCs can be employed to not only visualize cellular components but also real-time dynamic biological processes. For bioimaging applications, properties such as bright luminescence, photostability, long luminescence lifetime, emission in the NIR range and large Strokes shift are desirable. Although semiconductor quantum dots (QDs) have been proposed as an optical probe for biomedical imaging due to their high fluorescence intensity and good photostability, their intrinsic cytotoxicity and self-aggregating phenomenon inside cells have severely limited their use for in vivo imaging. Another common category of optical probe, organic dyes, are also restricted by their relatively low photostability and luminescence lifetime. In view of the aforementioned, protein-templated metal NCs present themselves as an attractive alternative. Their excellent chemical stability and photostability, together with their inherent biocompatibility, have made protein-templated metal NCs an emerging category of optical probes for both in vitro and in vivo bioimaging.

#### 5.2.1. In Vitro Imaging

Among the various types of metal NCs, biomolecule-protected metal NCs with biological activity are particularly suitable for bioimaging. The synergistic effect of the biological function of the biomolecule shell and the fluorescence properties of metal NCs facilitate the subcellular localization of the NCs for cell imaging. The adoption of fluorescent NCs for clinical applications had been hampered by a complicated synthesis methodology and the lack of selectivity towards the target of interest. However, Pan et al. successfully demonstrated that methionine stabilized gold NCs (Met-AuNCs) are stable and had high specificity towards cancer cells (Hela, MCF-7, HepG2, A549) [163]. The met-AuNCs showed bright fluorescence during in vitro imaging of the cancer cells but had no fluorescence detected in normal cells (WI-38 and CHO). The specificity of the NCs was attributed to the specific recognition of *L*-type amino acid transporters that are overexpressed in cancer cells. Methionine is an essential amino acid found in the primary sequence of proteins. This work hints that protein-templated metal NCs may also possess a certain degree of specificity for in vitro cell-based studies. Recently, Chakraborty et al. successfully synthesized HSA templated gold NCs (HSA-AuNCs) that can be used as a fluorescence probe to detect breast cancer cells [136]. The HSA-AuNCs were found to localize predominantly in the cytoplasm of the cancerous cell line (MDA-MB-231) but had no uptake in the normal cell line (MCF10A). Interestingly, upon further investigation, the group discovered that when the MDA-MB-231 cells were additionally incubated with Hg^2+^ ions, the fluorescence gradually declined over time. The time-dependent fluorescence quenching in a biological medium (MDA-MB-231) demonstrated its potential application as an in vivo sensor of Hg^2+^ ions.

**Figure 9:**
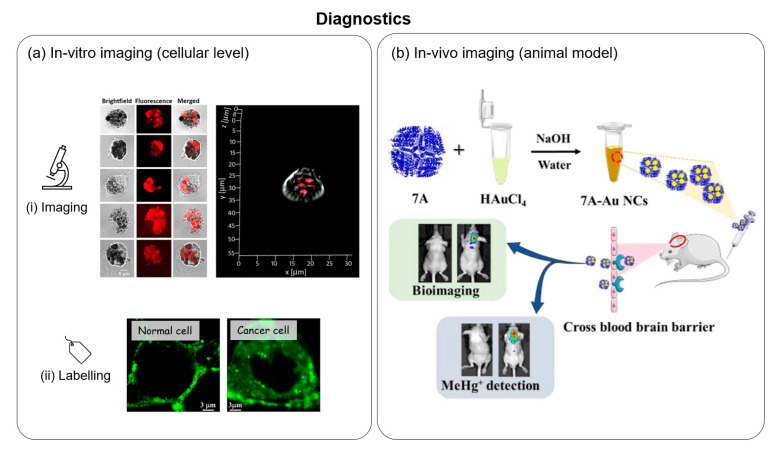
(**ai**) Confocal fluorescence microscopy images of dendritic cells after incubation with G0-OVA-AuNCs (left). Z-stack image of a single dendritic cell showing internalization of G0-OVA-AuNCs inside the cell (right). Reproduced with permission from [165]. (**aii**) Confocal image of normal breast cell (MCF10A) and breast cancer cell (MCF7) stained with BSA-templated Au-NC. Reproduced with permission from [166]. (**b**) Schematic illustration of the synthesis of the 7A protein-templated AuNCs (7A-Au NCs) and its applications in bioimaging and detection of MeHg^+^ in the mice brain. The 7A protein served as a biotemplate for the synthesis of AuNCs and conferred the NCs the ability to transverse the blood–brain barrier. In the presence of MeHg^+^, the bright fluorescence of the AuNCs can be quenched in vivo. Reproduced with permission from [59].

Due to the presence of abundant functional groups, proteins can be easily functionalized with biorecognition moieties to endow protein-templated MNCs with specific biological activity that can be exploited for bioimaging applications. For instance, Brzezicka et al. synthesized neoglycoprotein-protected gold NCs by conjugation of ovalbumin (OVA) with N-glycan G0 via the lysine residues or the N-terminus. The functionalization of OVA with glycan was previously reported to enhance the targeting of dendritic cells (DCs) and its subsequent uptake [164]. The prepared G0-OVA-AuNCs was then successfully demonstrated for application in in vitro imaging of DC [165]. As shown in Figure 9(ai), the uptake of G0-OVA-AuNCs by DCs produced intense fluorescence which allowed its imaging and visualization.

In the area of clinical diagnosis, cell labeling to differentiate between cancerous and non-cancerous cells is critical for the early detection and prevention of diseases. AuNCs that are templated by globular protein (HSA and BSA) were demonstrated to be able to discriminate between cell lines that are cancerous and non-cancerous [136,166]. In such applications, the uptake of the fluorescent NCs was significantly higher in cancer cells, thereby forming the principle through which cancerous cells can be differentiated. As shown in Figure 9(aii), the BSA@AuNCs accumulates primarily in the cell membrane for normal breast cells (MCF10A), whereas both the membrane and cytoplasm are stained in the case of the breast cancer cell (MCF7). The higher uptake of the AuNCs was attributed to the increased levels of glutathione in cancerous cell lines [167]. The fact that cellular glutathione is several-fold higher in cancer cells has been long established [168]. As glutathione is a stabilizing ligand for AuNCs, it was suggested that the glutathione in cancerous cells may replace the original BSA ligand, giving rise to a greater degree of internalization of AuNC in cancer cells. Apart from the labelling of cancer cells, MNCs have also been applied to the labelling of the nucleus [169], etc.

#### 5.2.2. In Vivo Imaging

For in vivo imaging applications, protein-templated metal NCs present themselves as an attractive fluorescence probe candidate due to their biocompatibility as well as excellent aqueous solubility and photo-/colloidal stability as compared to cytotoxic quantum dots and organic dyes which suffer from photobleaching. When using metal NCs as an imaging agent in vivo, some additional considerations include efficient renal clearance and effective accumulation in the target (e.g., tumors and cancer cells). The use of metal NCs for the in vivo imaging of tumor and cancer sites has been widely reported [170,171,172,173]. Interestingly, Lv et al. recently reported for the first time the use of 16-Mer ferritin-like protein (7A) as a template for synthesis of AuNCs [59] (Figure 9b). A unique feature of the 7A protein is its ability to transverse the blood–brain barrier. Hence, the as-prepared 7A-AuNCs were successfully applied to the in vivo imaging of mice brains for the detection of methylmercury (MeHg^+^), a deadly neurotoxin that accumulates in the brain. The detection was based on the fluorescence quenching effect in the presence of MeHg^+^.

Although much progress has been made in the bioimaging applications of metal NCs, most of them are based on the wavelength of light ranging from the visible light range to near-infrared I (NIR-I) (400–900 nm). This leads to some challenges as this range of wavelengths does not have high penetration depth in living tissues. Additionally, some biological tissues undergo auto-fluorescence in this range as well leading to unwanted interferences that affect the MNCs bioimaging system and worsen the signal-to-noise ratio. Hence, imaging in the NIR-II region (1000–1700 nm) has been receiving much attention [174,175,176,177]. For instance, Wang et al. engineered gold NCs stabilized by ribonuclease-A (RNase-A) to achieve red-shifted emissions in the NIR-II region at 1050 nm and with a QY of 1.9% [177]. The engineered RNase-A@AuNCs displayed excellent sensitivity in gastrointestinal imaging performed on mice models and was superior to previously reported silver-based or rare earth NIR-II emitters. Apart from bioimaging applications, it was suggested that the deep tissue penetration ability due to fluorescence imaging in the NIR-II shows promise in fluorescence-guided surgery applications.

Apart from fluorescence imaging, various other imaging techniques such as photoacoustic (PA) imaging, computed tomography (CT), positron emission tomography (PET) and magnetic resonance imaging (MRI) have been employed for bioimaging [178,179]. Each imaging technique has its own pros and cons. For instance, in the case of fluorescence imaging, one severe limitation is the penetration depth of light as described earlier. Multimodal imaging is thus devised to integrate various imaging techniques, which overcomes the limitations of each individual imaging technique and combines the advantages of each imaging method. In this vein, luminescent metal NCs can be integrated with other techniques such as PA, CT and PET to develop a multimodal imaging system with superb sensitivity and resolution. For instance, Yang et al. successfully developed multifunctional fluorescent gold NCs stabilized by milk metalloprotein alpha-lactalbumin that can not only be used for the local bioimaging of tumors but also whole-body magnetic resonance imaging (MRI) and X-ray computed tomography (CT) for detection of breast cancer [173]. It is also worth pointing out that the developed gold NCs are ultrasmall, facilitating renal clearance, and are made up of clinically validated biocompatible proteins. All of these are critical factors for the translation of such technologies from the laboratory to real-world applications. In a separate study, Zheng et al. successfully synthesized gallium-68-doped, glutathione-templated AuNCs for tumor detection [172]. As gallium-68 is a radioisotope used in nuclear medicine, the gallium-68-labelled gold NCs can be employed for tumor detection via PET/CT imaging.

### 5.3. Drug/Gene Delivery

The ability to target specific organelles or cells is a major consideration in drug delivery therapies. The recognition capability can often be achieved through the rational selection of the template molecule [130,180]. Protein-templated MNCs can easily be conjugated with chemotherapeutic drugs through the rich functional groups on protein for effective drug delivery. One major concern in using chemotherapeutic drugs is their cytotoxicity towards both healthy and cancerous cells. Another issue is their short lifetime in the human body as they are quickly metabolized and cleared out of the human body system by the kidney and liver. However, protein-templated metal NCs can resolve these limitations through their inherent biocompatibility and ease of functionalization to accumulate preferentially in tumor sites. For example, doxorubicin (DOX), an anticancer drug, was conjugated with BSA-AuNCs that was modified with folic acid [181]. The folic acid provided the specific binding towards overexpressed folate receptor in tumors. The as-prepared NCs simultaneously displayed excellent tumor targeting efficacy and anti-tumor activity, highlighting its potential for use in tumor imaging and therapy. Several other groups have also reported on conjugating DOX on NCs for cancer therapy [182,183,184,185].

**Figure 10:**
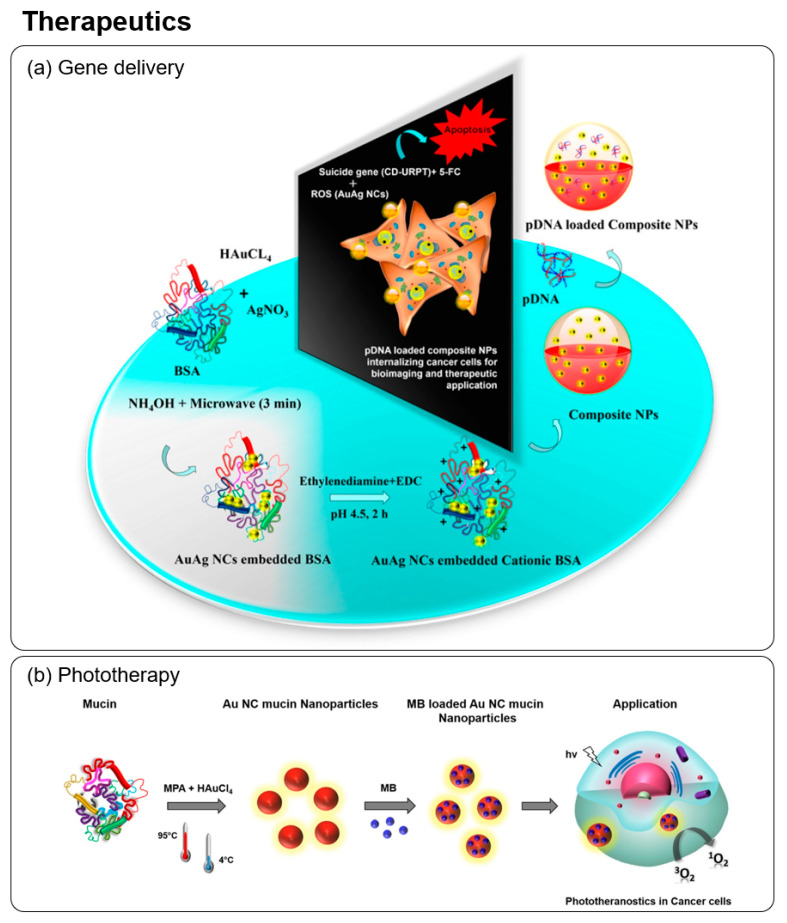
(**a**) Schematic representation showing the synthesis of cationic BSA templated Au-Ag NCs composite nanoparticles for suicide gene delivery and bimetallic NCs-induced ROS generation in cancer cells. The composite nanoparticles can simultaneously be used for bioimaging to track the delivery of the suicide gene into the cells. Reproduced with permission from [187]. (**b**) Schematic illustration of the synthesis steps of MB-loaded Au NC-mucin nanoparticles and its application in photodynamic therapy of cancer cells. Reproduced with permission from [188].

Apart from drug delivery, nanoparticles can also be used as a carrier for gene delivery to achieve beneficial therapeutic outcomes by counteracting or repairing defective genes in the cells that cause diseases [186]. Dutta et al. developed a BSA-embedded bimetallic NCs system for theranostic gene delivery to cancer cells [187]. As shown in Figure 10a, the synthesized cationic BSA-Au/AgNCs were fabricated into composite nanoparticles to be loaded with pDNA which encodes for the suicide gene CD-UPRT. The composite nanoparticles triggered a therapeutic response by converting prodrug 5-FC to 5-FU and leading to a generation of reactive oxygen species (ROS), both of which led to apoptosis cell death.

### 5.4. Phototherapy

Photodynamic therapy (PDT) and photothermal therapy (PTT) are techniques in phototherapy in which the light of specific wavelengths is used in the treatment of diseases such as cancer and tumor cells non-invasively. Both involve the use of photosensitizers (PSs), which upon irradiation are capable of transferring the absorbed photon energy to heat energy or surrounding oxygen molecules, leading to the formation of ROS (e.g., free radicals or singlet oxygen) [189]. The generation of ROS can trigger cell death, resulting in the treatment of diseases such as cancer. Both protein-templated monometallic and bimetallic (Au/Ag) NCs have been reported to show PDT effects [45,190]. Recently, Dutta et al. developed mucin-embedded AuNCs that were loaded with PS—methylene blue (MB) for photodynamic therapy in cancer cells [188]. As illustrated in Figure 10b, the synthesized MB-loaded mucin-AuNC was internalized in the cancer cells and, upon irradiation with 640 nm light, led to the increased formation of singlet oxygen and the apoptosis-mediated cell death.

PTT is a potent and noninvasive therapy that has been employed as a treatment for cancer as compared to the traditional methods of chemotherapy, radiotherapy and surgery [191]. It has been suggested to thermally induce damage on the integrity of cell membrane, leading to the eventual death of cancer cells. One advantage of PTT is its use of an external laser irradiation that allows for tumors to be targeted specifically while minimizing the harm to nearby healthy cells. Protein-templated metal NCs have been found useful for PTT in two means. First, the protein-templated synthesis protocol of noble metal can be extrapolated to synthesize NCs of PTT active components. For example, protein-templated Gd NCs have been synthesized for PTT with multimodal imaging ability [192]. In a separate study, Pan et al. assembled gold nanorods which are PTT active with iron oxide nanoparticles and gold NCs within the BSA protein to create a plasmonic and magneto-luminescent multifunctional nanocarrier that can be used for both magnetic targeting and plasmonic PTT [193]. Furthermore, Zhou et al. synthesized Gd^3+^ and CuS-coloaded small bovine serum albumin nanoparticles (GdCuB) that can be employed for MRI-guided PTT [194].

## 6. Conclusions and Future Perspective

This article reviews the recent advancement of a special type of metallic nanoparticles with core size less than 3 nm and protected specifically by proteins, i.e., protein-templated metal NCs. Protein-templated metal NCs feature intrinsic biocompatibility and intriguing luminescence properties, which are of particular interest in biomedical applications. This review summarizes the four important aspects of protein-templated metal NCs, including their synthesis approaches, physicochemical properties, characterizations and biomedical applications. Specifically, the molecular structure of protein determines the ease/hardness of synthesis of metal NCs, leading to different sources of reducing ability. The luminescence generated by light irradiation or electrochemical reactions is the most interesting attribute of the protein-templated metal NCs. Analytical techniques such as matrix-assisted laser desorption ionization time-of-flight (MALDI-TOF) and circular dichroism (CD) form the ideal characterization techniques to better understand the formation process of photoluminescent protein-templated metal NCs and the structure change of protein corona. Two case studies are featured on how information obtained from different characterization techniques that are combined to disclose valuable insights into the formation of photoluminescent protein-templated metal NCs and how the protein structure change information can be used to control the formation kinetics and design interesting applications. Various important applications of protein-templated metal NCs including biosensing, diagnostics, drug/gene delivery and phototherapy prove the great potential of the protein-templated metal NCs. Although we have witnessed ever-growing interests in the synthesis and biomedical applications of protein-templated metal NCs in the past 10–15 years, there are still some important aspects of protein-templated metal NCs pending for further elucidations. First, the photoluminescence quantum yields of protein-templated metal NCs are still behind those of organic dyes and semiconductor quantum dots (typically PLQY < 10%, as summarized in Table 1 and Table 2). New synthesis routes and/or post-treatments are needed to further boost the PLQY of protein-templated metal NCs to a level competitive to organic dyes and semiconductor quantum dots. Second, fully resolved structures of protein-templated metal NCs are yet to be elucidated in spite of the fact that many proteins have known structures that are available in the Protein Data Bank (https://www.rcsb.org/) (accessed on 27 June 2023). Nevertheless, no single crystals of protein-templated metal NCs have been obtained until today. Thirdly, the sizes of protein-templated metal NCs are solely determined by MALDI-TOF. Mass spectra with isotopic resolution of protein-templated metal NCs are not achieved yet, possibly due to the abundance of functional groups from the surface protein corona, which may rely on more advanced characterization techniques emerging to solve this problem. Last but not least, how protein-templated metal NCs interact with the biological system is still not fully understood. A deeper understanding of the mechanism is the prerequisite to bring the use of protein-templated metal NC to a much higher clinically viable level. Despite these current hurdles and challenges, we believe that the area of protein-templated metal NCs will continue to be prosperous and hope that this review article could serve an updated, complete and interesting reading material for anyone who is interested in this exciting field.

## Figures and Tables

**Table 1:** Summary of the synthesis approaches of protein-templated metal nanoclusters and their physical properties/applications (Protein examples are sorted in alphabetical order).

Synthesis Approach		Protein Template	Protein Size	Metal	Photoluminescence Property (λ_ex_/λ_em_, QY)	Size (nm)	Application	Ref.
Intrinsic reducing power of protein	Triggered by alkaline pH	Human serum albumin	66.4 kDa	Cu	325 nm/405 nm	-	Assay for detection of human serum albumin	[53]
	Tyrosine residues in lysozyme activated by high pH	Lysozyme	14 kDa	Au	300–450 nm/657 nm, 5.6%	1	Sensing of Hg^2+^	[54]
	Reduced by carboxyl groups in histidine/tyrosine	Lysozyme type VI	-	Au	380 nm/455 nm, 56%	-	Sensing of glutathione	[55]
	Reduced by tyrosine residues at alkaline pH; reduced by carboxyl groups in acidic conditions	Pepsin (porcine)	3.4 kDa	Au	Au_25_: 360 nm/670 nm, 3.5% Au_13_: 330 nm/510 nm, 5.0% Au_5_, Au_8_: 330 nm/402 nm, 3.7%	1–2	Sensing of Pb^2+^ and Hg^2+^	[56]
	Activated by alkaline conditions	Papain	23.4 kDa	Au	470 nm/660 nm, 4.3%	1.2 ± 0.2	Sensing of Cu^2+^	[57]
	Activated by alkaline conditions	Trypsin	5 kDa	Au	520 nm/690 nm, 6.5%	2.7 ± 0.4	Sensing of heparin and in vivo cancer imaging	[58]
	Activated by alkaline conditions	16-mer ferritin-like protein	<100 kDa	Au	500 nm/650 nm	2	Sensing of Hg^2+^ and in vivo bioimaging	[59]
Addition of reducing agent	NaBH_4_	Bovine serum albumin	66.5 kDa	Ag	480 nm/625 nm, 0.4%	1.7	Photodynamic therapy	[45]
	Ascorbic acid	Bovine serum albumin	66 kDa	Cu	330 nm/400–600 nm	2.3 ± 0.4	Antifouling	[60]
	N_2_H_4_.2H_2_O	Bovine serum albumin	66.5 kDa	Cu	525 nm/643 nm	~2.5	Sensing of creatinine	[61]
	NaBH_4_	Keratin	40–68 kDa	Ag	400 nm/705 nm, 1.7%	2.53 ± 0.54	Sensing of Hg^2+^	[62]
	N_2_H_4_	Lysozyme	14 kDa	Cu	490 nm/510 nm, 18%	3–5	Cellular imaging	[60,63]
	Dithiothreitol	Lysozyme	14.3 kDa	Cu	360 nm/640 nm, 6.1%	1.5 ± 0.31	Sensing of Cu^2+^ and Vitamin B12	[61,64]
	N_2_H_4_	Thunnus albacares fish protein	-	Cu	330 nm/446 nm	1–2	Sensing of Fe^3+^	[65]

## Data Availability

Not applicable.
